# Do We Mean What We Say?

**Published:** 1885-04

**Authors:** Ausburn Towner


### DO WE MEAN WHAT WE SAY?

AUSBURN TOWNER.

Our language is of such peculiar construction, that if we listen to the commonest conversation, or think about the words we ourselves use, we must assuredly be impressed with the thought that very frequently we can hardly mean what we say, or we cannot mean what the expressions we use really mean. From earliest infancy and childhood we are accustomed to such perversions of our tongue and they therefore fall with less effect upon our maturer ears. When we see a mother hugging her baby to her bosom with that delight which only a mother can feel and hear her exclaim: "Oh! you are too sweet for anything, mudder could eat up her darling," or "She could hug her moss a' death," or when taking another tack over the shortcomings of the child, she cries out, not very elegantly, but with a similar amount of extravagance and exaggeration: "Oh! you naughty, naughty imp! You ought to be skinned alive," it is fair to presume that in neither instance does she mean exactly what she says. Yet the child, early in life, used to such expressions, grows into a somewhat similar way of talking, and is not disturbed by hearing it among his companions.

It might be said, and it would be borne out, I think, by examination, that our whole language has been built up by just such perversions and exaggerations. Our commonest salutations, "How do you do?", "Good morning," or "Good morrow," "How's your health" and the like, with similar greetings in other tongues as "Wie gehts?" in the German, have, in reality, no meaning at all, and applied as salutations, appear, when closely regarded, just as absurd as the mother's talk to her infant. "How do you do?" "Do what?" might be asked in return, conscientiously and without meaning to joke, or: "Just as I have a mind to," as I once heard in reply to the greeting. "Good morning," is said whether the sunshines or the rain falls, perhaps meaning in either case that the morning is good in some directions, no matter how unpleasant it may be in others. "How's your health?" or "How d'ye feel?" is as frequently applied to one who is robust and vigorous as it is by a doctor or to an invalid.

Our language is singularly poverty-stricken in this respect. There is only one word that fully fills the requirements of greeting the appearance of a friend. That is "Welcome!" It means precisely what you want to express, that he who is before you is "well come," or it is well that he has come. Of words properly expressing a pleasant salutation when you casually meet a friend, the English language has none, and those used are absurd, for in none of them do you mean what you say.

So do our parting salutations show a similar poverty in so far as the matter of going direct to the sentiment required is concerned. "Farewell" or "Fare thee well," which is the same, and could, just as well, be spoken as a welcome greeting as a parting salutation. "Good-bye," merely a contraction of "God be with you," is a good wish at any time and under any circumstances, and "Adieu" or "to God," is rather an intimation of a recommendation for one who is soon to appear before the Creator, and in this sense is assuredly not a very pleasant reminder. The senseless and absurd character of these two latter expressions, may be shown in the fact that notwithstanding their very apparent meaning, I have heard them used with great unction by persons who denied the existence of a God! They, most certainly, did not mean what they said.

So the Yankee's frequent use of the expression: "I guess," or the Southerner's "I reckon" or "I expect," each for "I think" or "I believe," as well as the Englishman's "You know," are uttered when far from there being any guess work, it is a matter of affirmation; when there is no reckoning or expectation in the matter at all, but certainty and realization and when the person addressed doesn't really know at all what it is asserted he knows.

It is said that an Englishman, himself addicted to the colloqualism mentioned, undertook to correct a countryman for using it, and in doing so, mixed himself up as follows: "No, sir, you know, you say you know,—you know, but we don't know,—you know." There was a distinguished president of a college who will be remembered by a number of Elmirans, who had the equally ridiculous habit of prefacing almost every sentence he uttered, with the words: "I say." An under graduate, having mimicked the doctor, the matter came to the knowledge of the latter and he sent for the young man to rebuke him, beginning thus: "I say, they say, you say, I say, I say,"—when the ridiculous nature of his sentence broke in upon him and the youth was sent away without his reprimand.

Much of this kind of expression, when it is not purely a personal habit, arises from the extreme elasticity of the English language. Words twisted from their normal meaning from some noted incident to which they are applied, or constantly used in connection with some conspicuous event, have new applications in the mouths of the common people. To that generation they are "slang," but mayhap, to the next, they become good English and find a place in the dictionaries, the foolishness of one generation, as it is often claimed, becoming the wisdom of the next. I remember in my boyhood, there was an expression: "over the left," that was considered particularly vulgar and sedulously barred from all decent society, although it was a most vigorous sentence we thought. To my surprise, I observed it in a dictionary of high authority recently, with the definition given it as "on the contrary." It meant more than that in our vocabulary. Whence its origin, who can tell? I would not be at all surprised now to hear it some day in the pulpit, or see it in a work of the best taste. There are hopes, in the light of such a fact, of the permanence and ultimate high character of such expressions as "played out," "I should snicker," "you just bet," and other similar curbstone utterances, whose real meaning is very far from their assumed one.

There are constantly appearing such words and terms, many of them finding it rather a difficult matter to become naturalized, others however, making out to stick. We should be thankful that our language did not become permanently burdened with such words as Loco foco, Hunker, Barnburner, Copperhead, Stalwart and Half-Breed, each one a term wrested from its inherent and well-established meaning to designate something very temporary in its nature. It is to be hoped that the manufactured term "Mugwump," whose meaning no one seems able to set forth, will meet with a similar fate. When such a deformed monstrosity as that is deliberately adopted into our already large family of words, there should be a vigorous protest raised in some direction.

There is one instance of the complete change of a word that is rather peculiar. This is the term "Gossip." We do not give it a very elevated place in our vocabulary, readily agreeing with the dictionaries which put it down as meaning an "idle tattler" or "tale-bearer." It really means "a covenant with God," and was the name given to a sponsor or god-father by our Anglo-Saxon forefathers. Junius tells how it acquired its present designation. "Female gossips," he says, "under cloak of this spiritual relationship, used to meet to tell stories and tipple over them." It can be seen how quickly and easily, the meaning of the term could have changed, by the behavior of a few who unworthily occupied the positions designated. How long did it take to manufacture the verbs "to burgle," and "suicided," both of them outrages on the English language, and yet gradually and surely becoming a part of it, out of the substantives burglars and suicide?

I heard, not long since, with considerable astonishment, an intelligent and cultured legal gentleman, in speaking of the distribution of an insolvent debtor's estate, *pro rata*, say: "After certain proceedings, the property would be *pro rated!*" Yet I suppose even that, will, some time or other, become at least good "Law English," which is as dreadful in some aspects, as good "Law Latin."

Manufacturing new words from combinations of words in the same language, is not always the best way of increasing or improving our vocabulary. Of course, if it is done and the new combination is necessary, its constant use will soon make its meaning well-known, but it is often cumbersome and more than likely does not express the meaning it is intended to convey. Take the word steamboat. We all know what it means now, but supposing it was presented to us for the first time, would we not rather consider it a boat of steam rather than one propelled by steam? Take words on the contrary derived from other tongues. Telegraph for instance or telephone, the first from the Greek, meaning a "writing from afar," the latter "hearing from far off." They express the notions desired to be conveyed precisely and have, besides in their meaning, a poetry, to which not even science is averse.

There is another reason why in our every day talk, we do not always mean precisely as we say, the inclination of the human mind to adopt a figurative style when speaking of anything new; when in passion or excitement or when in rhetorical or poetical flights. How it takes us back to the early days of the human race when names were being given to all the objects that came within their view, to be told that when Stanley, in the interior of Africa, showed the natives an umbrella, they had no term to apply to it, except that which, in their language meant "a cloud." Surely, a picturesque designation, but hardly more so than our own, however, "umbrella," which comes from the Latin "umbra" meaning "a shadow" or "shade." The word is also the root of "umbrage," which has singularly come to mean, not only the shade or shadow cast by trees, but also figuratively, a resentment arising from a suspicion of an injury. Curiously enough too its adjective, "umbrageous," has no such double meaning, its application being exclusively applied to the foliage. Why could we not, as well, say of one who felt a resentment toward another for some supposed injury, that he was an "umbrageous fellow?" Terms or expressions, equally as figurative, cease to appear so, after a time, their literal sense having been lost in the habit of applying them differently. We perhaps never think of it, but we can hardly mean it when we say of a rider falling that "he bit the dust." He does nothing of the kind. Or of a passionate man that "he was boiling over with anger," when we know he is not; or of even a railroad train that it is as "swift as the wind." When we think for a moment over these expressions, or others similar to them that can be easily recalled, we are apt to smile a little at their incoherence, although from constant use they seem all right. How absurd it seems to read that some one "lifted up his voice and wept," as though his speaking medium was some ponderable substance; or of another that "his heart leaped to his mouth," a species of anatomical gymnastics that never occurred, or that, "She dropped her chin upon her bosom and remained silent," which suggests a rather long jaw, and accounts for her not speaking; or that "She slowly rolled her eyes around," an amusement that would more than likely be the precursor of blindness. In the Drawer of a late number of Harper's *Magazine* occurs the peculiar statement that "A well-known artist in London having dined sumptuously upon a bill of fare, &c." It was meant in earnest, but the dinner must have been taken with his eyes rather than his stomach, for an ordinary conception of a "Bill of Fare" is rather a description of the eatables than the eatables themselves. In still another view of the habit. When a member of a deliberative body solemnly rises and says:

"I move you, sir, that this resolution be laid on the table," how every one would laugh if, instead, he had said: "I stir you, sir, that this resolution, &c.," yet "move" and "stir" have almost precisely the same meaning originally and inherently. Or when some one says: "I cordially endorse the action of my friend," meaning to say that he approves it, supposing he had said instead: "I write upon the back of the action of my friend," for that is precisely what endorse means, what a shout there would be. He "backs" the action, to be sure in a strong way, or stands at his friend's back, and thus in a figurative way endorses him and it. Or, when a member of our common council moves to "adopt" the report of a committee does he really mean what he says? What he really means is to make the action of the committee the action of the council. What he says means literally "to take another's child as one's own," for that is to adopt. It has been a long and rounabout figurative journey that the word has taken from the time when it was a frequent action in the old Roman days to these when it has come to mean to take the "children of another's brain as one's own." Perhaps it was at first used in this way by some witty fellow anxious to perpetrate a pun.

I have often been amused at the efforts of some well-meaning persons, mostly women, to be precise and elegant in the use of language, skipping such solid, sound words as "folks" and "victuals," and saying instead "people" and "food." "People" don't mean "folks" by any means, and the term can not be ground down yet awhile into such a diminutive place. It means a nation or a race, not a family, nor a mere company. You might call the English Queen Victoria's "people," but you would hardly call her family by that term, any more than you would or should call any one's family thus.

Such inquiries as this in which I have been engaged, are full of interest and amusement and offer a wide field for one who has the patience and is possessed of a good dictionary. Instances of which I have named only a few, similar in most respects, worse in many, may be found in about every book that is printed and certainly in every newspaper. We may deplore them and wish that our language was more permanent and less liable to changes that make it uncertain as to its exact meanings, but it is beyond the power of lexicographers, etymologists or any set of men to control it. It is a living, moving monster that has already nearly conquered the world and laid all tongues tributary to it. What it wants, it takes, without regard to law or custom; what it don't want it won't have under any consideration. Its selections have no regard to sound, sense or construction, a pretty word being as likely to be thrown aside, as an ugly one to be chosen and appropriated. Perhaps we should be content with the notion that in a majority of in stances, and for the common every day work of the world, we are able to so express ourselves that our meaning may be clear enough for all purposes, forgetting that very likely our descendants in the century or so to come would be unable to understand us; that no sentence was ever uttered but could have more than one construction and, that it is claimed by the wisest, that language, at the best, was invented only to conceal our thoughts.

